# Plasma level of peroxiredoxin 3 in patients with polycystic ovarian syndrome

**DOI:** 10.1186/s12902-019-0358-3

**Published:** 2019-03-14

**Authors:** Hou-Li Liu, Tian-Tian Li, Ai-Qun Yu, Jingmin Li, Xiaoyan Li, Lianqin Li, Tomonori Kaifu

**Affiliations:** 1 Department of Laboratory Medicine, Yantai Affiliated Hospital of Binzhou Medical University, Yantai, 264100 China; 2 Department of Reproductive Medicine, Yantai Affiliated Hospital of Binzhou Medical University, 717 Jinbu Street, Muping-district, Yantai, 264100 China; 3 Institute of Psychology, Chinese Academy of Sciences, Beijing, 100101 China; 4 Graduate School of Chinese Academy of Sciences, Beijing, 100049 China; 5 School of Basic Medical Sciences, Binzhou Medical University, Yantai, 264003 China; 6 Division of Immunology, Faculty of Medicine, Tohoku Medical and Pharmaceutical University, 4-4-1, Komatsushima, Aoba-ku, Sendai, 981-8558 Japan

**Keywords:** Peroxiredoxin, Reactive oxygen species, Oxidative stress, Polycystic ovarian syndrome, Insulin resistance

## Abstract

**Background:**

As a member of peroxiredoxin (PRX) family, PRX3 is predominantly located in mitochondria and plays an important role of free radical scavenging. Since a body of evidence demonstrated the involvement of PRX3 in insulin secretion, insulin sensitivity, and glucose metabolism, the present study was conducted to investigate the role of PRX3 in the pathogenesis of polycystic ovarian syndrome (PCOS) featured in insulin resistance.

**Methods:**

Enzyme-linked immunosorbent assay was performed to detect plasma PRX3 in PCOS patients and control subjects. Levels of reactive oxygen species (ROS) and oxidized PRXs were detected in mouse islet cells treated with gradient glucose.

**Results:**

We did not find significant difference of fasting plasma PRX3 between PCOS patients and controls. No association was noticed between fasting plasma PRX3 and fasting plasma glucose or insulin. After oral glucose tolerance test (OGTT), PCOS patients showed higher levels of both glucose and insulin as compared to controls. The plasma level of PRX3 was significantly increased at 2 h and began to fall back at 3 h of OGTT. There was a one-hour time lag of peak values between plasma PRX3 and insulin, and the plasma PRX3 at 2 h was positively correlated with the insulin level at 1 h of OGTT of PCOS patients. In addition, the level of ROS was significantly elevated at 1 h and oxidized PRX3 was increased dramatically at 2 h of 16.7mM glucose stimulation in mouse islet cells.

**Conclusion:**

It seems that PRX3 does not show its antioxidant function under baseline conditions. Instead, PRX3 responds to oxidative stress induced by rapid increase of insulin and glucose in patients with PCOS.

## Background

Polycystic ovarian syndrome (PCOS) is one of the most common female endocrine disorders affecting about 5–10% women of reproductive age [1]. The clinical and biochemical features include oligo/anovulation, clinical/biochemical hyperandrogenism, and polycystic ovaries. In addition, insulin resistance (IR), hyperinsulinaemia, hyperglycaemia, glucose intolerance, dyslipidaemia, and obesity are frequently found in PCOS patients [2, 3]. Although the exact etiology of PCOS remains to be unclear, oxidative stress has been associated with the pathophysiology of the disorder [4, 5], which even passes to the next generation [6]. Peroxiredoxin (PRX) is a kind of antioxidant enzyme that reduces hydrogen peroxide (H2O2) into water using its conserved cysteines. PRX3 is mainly located in mitochondria and scavenges about 90% of mitochondrial H2O2 [7], playing a critical role for mitochondrial homeostasis [8]. We previously employed PRX3-knockout mice to investigate the functional role of PRX3. Our study indicated that PRX3 was an indispensible responder to oxidative stress in inflammation, pregnancy, and malignancy [9–11]. In 2011, Huh et al. reported the occurrence of obesity of PRX3-knockout mice and mitochondrial impairment of adipocytes such as increased oxidative stress, decreased mitochondrial biogenesis, and dysregulation of enzymes involved in glucose/lipid metabolism [12].

The involvement of PRX3 in glucose metabolism could be traced back to 2008 when Chen et al. reported the improvement of glucose tolerance by PRX3 through reduction of mitochondrial H2O2 [13]. Afterwards, considerable investigations demonstrated the role of PRX3 in glucose tolerance, insulin secretion, and IR. PRX3 protected pancreatic β cells from apoptosis to maintain insulin secretion [14]. More importantly, PRX3 plays a key role in maintaining mitochondrial homeostasis and increasing insulin sensitivity. PRX3-deficiency in mice exhibited metabolic disorders including impaired glucose tolerance, increased IR, and obesity. Further study showed elevated oxidative stress and down-regulation of fatty acid metabolism-ralated proteins in adipocyte, resulting in fat accumulation of PRX3-knockout mice [12]. Moreover, genetic variations or decreased expression of PRX3 were recognized in humans with obesity [12, 15]. Since a large proportion of patients with PCOS present obesity and IR, the present study is conducted to link plasma PRX3 with IR of PCOS patients.

## Methods

### Patients

After approval by the Ethics Committee of Binzhou Medical University (Yantai, China), we obtained written consent from all participants. The diagnostic criteria of PCOS were based on the revised Rotterdam consensus [16] including any two of the following three clinical features: (1) oligo/anovulation, (2) clinical and/or biochemical hyperandrogenism, and (3) polycystic ovaries on ultrasound. Women with other related disorders such as adrenal congenital hyperplasia, thyroid disease, Cushing’s syndrome, or androgen-secreting tumors were excluded.

### Definitions of obesity and calculation of IR

The body mass index (BMI) ≥28 was identified as obesity according to the diagnostic criteria for obesity in the Asia-Pacific region issued by World Health Organization (WHO) [17]. Homeostasis model assessment of IR (HOMA-IR) was calculated by fasting plasma glucose (FPG) × fasting insulin (FINS)/22.5.

### Oral glucose tolerance test (OGTT)

Fasting blood samples were collected from the antecubital vein of the subjects on the second or third day of the menstrual cycle. In addition to routine examination of reproductive hormones including follicle stimulating hormone (FSH), luteinizing hormone (LH), estradiol (E2), prolactin (PRL), and testosterone (T), a 75-g OGTT was performed to detect the changes of plasma glucose, insulin, and PRX3 at the time points 1 h, 2 h, and 3 h respectively. The plasma level of PRX3 was detected by enzyme-linked immunosorbent assay (ELISA). The kit for ELISA was purchased from NeoScientific (MA, USA) and the experiment was performed according to the manufacturer’s instruction. We assayed duplicates of each sample and measured the absorbance at 450 nm on a spectrophotometer.

### Glucose stimulation of mouse islet cells

The Beta-TC-6 mouse islet cell line was purchased from the Cell Bank of Chinese Academy of Sciences (Shanghai, China) and maintained in Dulbecco’s modified Eagle’s medium containing 15% fetal bovine serum, 100 U/L streptomycin, and 100 U/L penicillin. After obtaining enough cells, we re-cultured the cells in six-well plates. Two days later when the cell confluence reached more than 80%, we changed the medium as Krebs-Ringer Bicarbonate Buffer (KRBB: 129 mM NaCL, 4 .8mM KCL, 1 .2mM MgSO4, 1 .2mM KH2PO4, 2 .5mM CaCL2, 5 mM NaHCO3, 0.1% BSA, and 10 mM HEPES. pH: 7.4) containing 2 .8mM glucose and 2 μM 2′,7′-dichlorofluorescin diacetate (DCFH-DA, Molecular Probes). After incubation at 37 °C for 30 min, we applied KRBB containing glucose at 2 .8mM, 5 .6mM, and 16.7mM respectively. The level of reactive oxygen species (ROS) in cultured supernatants was estimated at the indicated time points through the oxidation of DCFH-DA using a fluorescent measurement system (Cytofluor 2350, Millipore, MA, USA) at 504 nm excitation and 524 nm emission. In addition, the level of oxidized two-cysteine PRXs in the mouse islet cells was detected by Western Blotting using rabbit polyclonal Anti-Peroxiredoxin-SO3 antibody (ab16830, Abcam, MA, USA).

### Statistical analysis

Software SPSS 20.0 was used for statistical analysis. The blood levels of glucose, insulin and PRX3 were compared between the two groups by *t* test. The rate of obesity between the two groups was compared by Chi-square analysis. The ROS level in cultured mediums of mouse islet cells was compared by analysis of variance. The association between plasma PRX3 and glucose or insulin was performed by Spearman correlation analysis. *P* < 0.05 was considered to be statistically significant.

## Results

### General information

We included fifty PCOS patients in the present study, and fifty-six infertile patients without PCOS were used as controls. As indicated in Table 1, there was no significant difference of age and FPG between the two groups. The levels of LH and T were significantly higher in PCOS patients than in controls. The obesity rate, the levels of BMI, FINS, and HOMA-IR were significantly higher in PCOS patients than in controls. We did not find significant difference of plasma PRX3 between PCOS patients and controls (Table 2).

**Table 1:** Comparison of reproductive hormones between PCOS patients and controls

	Age	FSH (U/L)	LH (U/L)	E2 (pmol/L)	PRL (nmol/L)	T (nmol/L)
PCOS	29.0 ± 3.0	4.45 ± 0.2	5.73 ± 0.4	129 ± 9.5	808 ± 62	1.71 ± 0.1
Control	30.8 ± 4.4	5.24 ± 0.2	3.62 ± 0.1	112 ± 5.5	670 ± 27	0.98 ± 0.04
*t* values	1.346	0.156	2.901	0.901	1.758	3.480
*P* values	0.197	0.877	0.006^a^	0.377	0.085	0.002^a^

^a^significant difference

**Table 2:** Comparison of general information between PCOS patients and controls

	Obesity (%)	BMI	FPG (mmoL/L)	FINS (mU/L)	HOMA-IR	PRX3 (ng/ml)
PCOS	52.0	28.9 ± 4.4	6.1 ± 1.2	18.7 ± 1.3	4.7 ± 0.2	29.5 ± 3.8
Control	10.7	23.2 ± 3.3	5.6 ± 0.5	11.2 ± 0.6	2.7 ± 0.1	29.3 ± 3.4
*t (χ*^2^*)* values	10.68	5.334	1.559	2.833	2.575	0.107
*P* values	0.001^a^	< 0.001^a^	0.119	0.005^a^	0.010^a^	0.915

^a^significant difference

To further understand the difference of plasma PRX3 between PCOS patients and controls, we sub-grouped the subjects according to the IR and BMI respectively. Subjects with IR ≥2.69 were divided into high IR sub-group, while the subjects with IR < 2.69 were divided into low IR sub-group. Obese PCOS patients with high IR (PCOS-IR^H^-Obese) were respectively compared to lean PCOS patients with low IR (PCOS-IR^L^-Lean) and lean controls with low IR (Control-IR^L^-Lean). As indicated in Table 3, there was no significant difference of plasma PRX3 between PCOS-IR^H^-Obese patients and Control-IR^L^-Lean subjects. The plasma level of PRX3 was slightly lower in PCOS-IR^L^-Lean patients than in PCOS-IR^H^-Obese patients but did not reach statistical significance.

**Table 3:** IR and BMI-based comparison of PCOS patients and controls

	n	Age	BMI	FPG (mmoL/L)	FINS (mU/L)	HOMA-IR	PRX3 (ng/ml)
Control-IR^L^ -Lean	28	29.6 ± 4.4	20.9 ± 3.3	5.3 ± 0.5	7.7 ± 0.6	1.8 ± 0.1	29.8 ± 3.4
*t* values		0.397	7.534	2.580	7.961	4.962	0.156
*P* values		0.695	< 0.001^a^	0.02^a^	< 0.001^a^	< 0.001^a^	0.877
PCOS-IR^H^ -Obese	22	29.3 ± 3.0	31.4 ± 3.1	6.7 ± 1.5	29.3 ± 10.5	9.2 ± 5.2	30.4 ± 3.6
*t* values		−1.093	4.315	2.661	6.001	4.646	1.154
*P* values		0.293	0.001^a^	0.02^a^	< 0.001^a^	0.001^a^	0.268
PCOS-IR^L^ -Lean	10	31 ± 2.6	24.8 ± 2.0	5.3 ± 0.4	9.0 ± 2.6	1.9 ± 0.5	25.1 ± 2.7

^a^significant difference

### Changes of plasma glucose, insulin, and PRX3 in OGTT

**Fig. 1:**
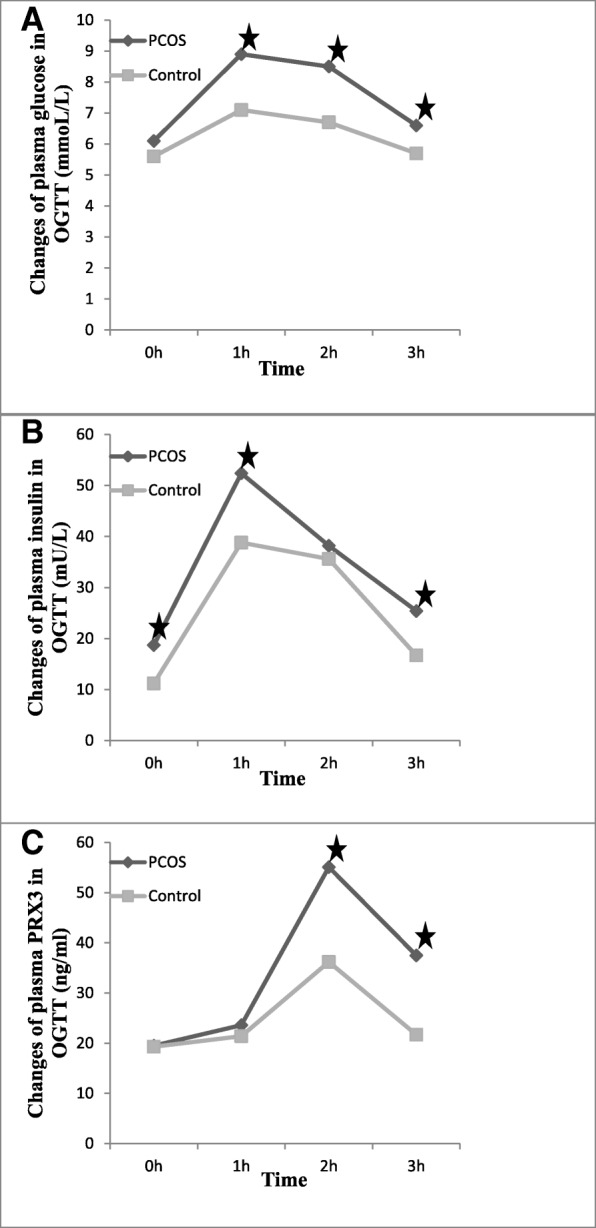
Changes of plasma glucose, insulin, and PRX3 in PCOS patients and controls undergoing OGTT. **a** Plasma glucose reached its peak at 1 h and began to fall back at 2 h. There was significant difference between PCOS patients and controls respectively at 1 h (*t* = 2.901, *P* = 0.006), 2 h (*t* = 2.578, *P* = 0.034), and 3 h (*t* = 2.772, *P* = 0.045). **b** The change pattern of plasma insulin was as the same as glucose in both PCOS patients and controls. The plasma insulin in PCOS patients was significantly higher than in controls respectively at 0 h (*t* = 2.833, *P* = 0.005), 1 h (*t* = 3.480, *P* = 0.002), and 3 h (*t* = 2.321, *P* = 0.046). **c** Plasma PRX3 reached its peak at 2 h (one hour later than the insulin peak) and began to fall back at 3 h. There was significant difference between the two groups at 2 h (*t* = 2.794, *P* = 0.012 and 3 h (*t* = 2.568, *P* = 0.039) respectively

We performed OGTT to understand the changes of plasma glucose, insulin, and PRX3 in PCOS patients. The plasma glucose in PCOS patients was significantly higher than in control patients at 1 h, 2 h, and 3 h (Fig. 1a), while the plasma insulin in PCOS patients was significantly higher than in controls at 0 h, 1 h, and 3 h respectively (Fig. 1b). As illustrated in Fig. 1c, the level of plasma PRX3 in PCOS patients reached its peak at 2 h (2 h vs 0 h: *t* = 4.421, *P* < 0.001) and began to fall back at the time point of 3 h (3 h vs 0 h: *t* = 3.560, *P* = 0.001). The plasma PRX3 in control subjects was increased at 2 h (2 h vs 0 h: *t* = 3.270, *P* = 0.002) and fell back to the level comparable to the baseline at 3 h (3 h vs 0 h: *t* = 0.570, *P* = 0.575). There was significant difference of plasma PRX3 between the two groups at the time points of 2 h and 3 h (Fig. 1c).

### The association of plasma PRX3 with plasma glucose and insulin

We applied the Spearman correlation analysis to understand the relationship of plasma PRX3 with plasma glucose and insulin. As indicated in Table 4, we did not find significant association of fasting plasma PRX3 with BMI, FPG, FINS, and HOMA-IR in either PCOS patients or control subjects. Interestingly, the plasma PRX3 at 2 h was positively correlated to the insulin level of 1 h in OGTT of PCOS patients (*r* = 0.451, *P* = 0.031).

**Table 4:** The association of fasting plasma PRX3 with BMI, FPG, FINS, and HOMA-IR

	BMI		FPG		FINS		HOMA-IR	
	*r* values	*P* values	*r* values	*P* values	*r* values	*P* values	*r* values	*P* values
PCOS	0.383	0.058	0.075	0.720	0.352	0.084	0.170	0.415
Control	−0.268	0.169	−0.056	0.778	0.207	0.292	−0.094	0.634

### Levels of ROS and oxidized PRXs in mouse islet cell

**Fig. 2:**
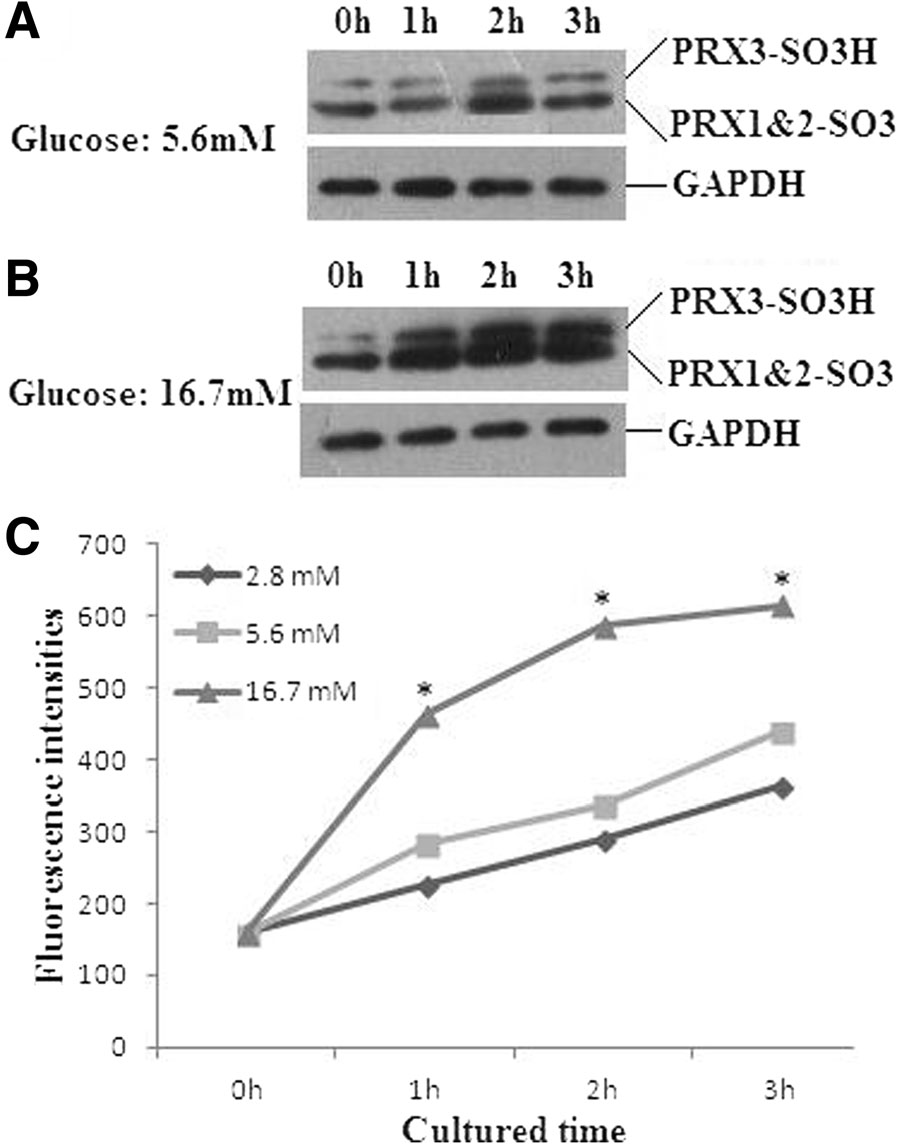
Levels of ROS and oxidized PRXs in mouse islet cells treated with glucose. **a** and **b** Oxidized PRXs were increased in a glucose concentration-dependent manner, and the signal for PRX3-SO3H was dramatically enhanced at 2 h of 16.7mM as compared to 5 .6mM glucose stimulation. **c** ROS production in mouse islet cells stimulated with 16.7mM glucose was significantly elevated at 1 h, 2 h, and 3 h respectively (*1 h: *F* = 4.641, *P* < 0.001; 2 h: *F* = 4.353, *P* < 0.001; 3 h: *F* = 3.460, *P* < 0.001). There was no significant difference in ROS production between 5 .6mM glucose stimulation and non-treated cells

To confirm the role of PRX3 in the response to insulin releasing, we detected the levels of ROS and oxidized PRXs in mouse islet cells treated with gradient concentrations of glucose. As shown in Fig. 2, oxidized PRXs were increased in a glucose concentration-dependent manner, and the signal for PRX3-SO3H was dramatically enhanced at 2 h of 16.7mM glucose stimulation as compared to 5 .6mM glucose stimulation (Fig. 2a and b). There was no significant difference in ROS production between 5 .6mM glucose stimulation and non-treated cells, while the ROS production in mouse islet cells stimulated with 16.7mM glucose was significantly elevated at 1 h, 2 h, and 3 h respectively (Fig. 2c).

## Discussion

In the present study, we examined the plasma level of PRX3 and did not find significant difference between PCOS patients and controls. However, the plasma PRX3 was dramatically increased in the insulin releasing test, especially in PCOS patients. There was a one-hour time lag of peak values between plasma PRX3 and insulin, and the positive correlation of plasma PRX3 with insulin suggests PRX3 to be an indicator of a short sharp release of insulin.

Although the etiology of PCOS has not been well known, IR and/or hyperinsulinemia are considered to play a key role in the pathophysiology of PCOS [18]. High levels of glucose and insulin can cause oxidative stress and have significant negative impacts on the mitochondrial homeostasis of islet beta cells [19, 20], forming a vicious circle in the pathogenesis of PCOS [21–23]. In the present study, we did not notice significant increase of fasting plasma PRX3 in PCOS patients. Since the plasma level of PRX3 was increased in the OGTT of both PCOS patients and controls, we suppose that PRX3 responds to the oxidative stress induced by rapid insulin release. Our in vitro detection showed elevated ROS and oxidized PRX3 in glucose treated mouse islet cells, which resembled the changes of plasma PRX3 in the subjects undergoing OGTT and provided strong support for the hypothesis. Another contributory factor for the elevated plasma PRX3 would be the existence of inflammatory state in patients with PCOS. Previous studies demonstrated high levels of inflammatory markers such as C-reactive protein or proinflammatory cytokines such as tumor necrosis factor α and interleukin-6 in peripheral leukocytes of PCOS patients, which contribute in turn to the induction of oxidative stress and IR [24, 25]. Furthermore, acute hyperglycemia stimulated an increase of ROS generation by leukocytes of PCOS patients [26, 27]. The present study showed higher plasma glucose in PCOS patients at 1 h, 2 h, and 3 h of OGTT as compared to control subjects. Increased plasma PRX3 might represent at least partially a response to an augmented oxidative stress of circulating leukocytes.

According to the previous reports, other PRX members including PRX1, PRX2, PRX4, and PRX6 respond actively to glucose and/or insulin-related oxidative stress [28–30]. In the present study using mouse islet cells, the oxidation of PRX3 was quite fewer as compared to that of PRX1 and/or PRX2 under basal conditions. However, oxidized PRX3 was significantly increased after glucose stimulation, along with the increase of oxidized PRX1 and/or PRX2. Another study conducted by Al-Masri et al. showed higher plasma levels of PRX1, PRX2, PRX4, and PRX6 in patients with type 2 diabetes than in control subjects. The plasma PRX1 in well-controlled diabetic patients fell back to the value comparable to that of non-diabetic controls, while PRX2, PRX4, and PRX6 remained to be at high level in both well-controlled and poorly-controlled diabetic patients [28]. These results suggested a cooperative response of PRX members to glucose-induced oxidative stress on the one hand and different responding patterns on the other. It seems that PRX2, PRX4, and PRX6 performed their antioxidant function in a routine manner, while PRX3 would emerge as a ROS scavenger under urgent or severe oxidative stress. In fact, most of the previous studies on the involvement of PRX3 in glucose metabolism/IR were conducted with several stressors or transgenic animals instead of “natural conditions” [13, 14, 27].

## Conclusions

In conclusion, the present results have provided evidence that PRX3 plays an essential role in the response to oxidative stress induced by rapid increase of glucose and insulin. Detection of plasma PRX3 would be useful for the evaluation of IR in PCOS patients.

## Abbreviations

BMI: Body mass index
DCFH-DA: 2′,7′-dichlorofluorescin diacetate
ELISA: Enzyme-linked immunosorbent assay
FINS: Fasting insulin
FPG: Fasting plasma glucose
H2O2: Hydrogen peroxide
HOMA-IR: Homeostasis model assessment of IR
IR: Insulin resistance
KRBB: Krebs-Ringer bicarbonate buffer
OGTT: Oral glucose tolerance test
PCOS: Polycystic ovarian syndrome
PRX: Peroxiredoxin
ROS: Reactive oxygen species
